# Cytopathic SARS-CoV-2 screening on VERO-E6 cells in a large-scale repurposing effort

**DOI:** 10.1038/s41597-022-01532-x

**Published:** 2022-07-13

**Authors:** Andrea Zaliani, Laura Vangeel, Jeanette Reinshagen, Daniela Iaconis, Maria Kuzikov, Oliver Keminer, Markus Wolf, Bernhard Ellinger, Francesca Esposito, Angela Corona, Enzo Tramontano, Candida Manelfi, Katja Herzog, Dirk Jochmans, Steven De Jonghe, Winston Chiu, Thibault Francken, Joost Schepers, Caroline Collard, Kayvan Abbasi, Carsten Claussen, Vincenzo Summa, Andrea R. Beccari, Johan Neyts, Philip Gribbon, Pieter Leyssen

**Affiliations:** 1 Fraunhofer Institute for Translational Medicine and Pharmacology ITMP, Discovery Research ScreeningPort, Schnackenburgallee 114, 22525 Hamburg, Germany; 2 Fraunhofer Cluster of Excellence for Immune-Mediated Diseases CIMD, Theodor-Stern-Kai 7, 60596 Frankfurt am Main, Germany; 3 KU Leuven, Department of Microbiology, Immunology and Transplantation, Rega Institute for Medical Research, Laboratory of Virology and Chemotherapy, Herestraat 49 - box 1043, 3000 Leuven, Belgium; 4 Dompé Farmaceutici SpA, via Campo di Pile, 67100 L’Aquila, Italy; 5 Dipartimento di Scienze della vita e dell’ambiente, Cittadella Universitaria di Monserrato, SS554, 09042 Monserrato, Cagliari Italy; 6 EU-OPENSCREEN ERIC, Campus Berlin Buch, Robert-Rössle-Str. 10, 13125 Berlin, Germany; 7 Department of Excellence of Pharmacy, University of Naples Federico II, Via D. Montesano, 49, 80131 Naples, Italy

**Keywords:** Mechanisms of disease, Viral infection

## Abstract

Worldwide, there are intensive efforts to identify repurposed drugs as potential therapies against SARS-CoV-2 infection and the associated COVID-19 disease. To date, the anti-inflammatory drug dexamethasone and (to a lesser extent) the RNA-polymerase inhibitor remdesivir have been shown to be effective in reducing mortality and patient time to recovery, respectively, in patients. Here, we report the results of a phenotypic screening campaign within an EU-funded project (H2020-EXSCALATE4COV) aimed at extending the repertoire of anti-COVID therapeutics through repurposing of available compounds and highlighting compounds with new mechanisms of action against viral infection. We screened 8702 molecules from different repurposing libraries, to reveal 110 compounds with an anti-cytopathic IC_50_ < 20 µM. From this group, 18 with a safety index greater than 2 are also marketed drugs, making them suitable for further study as potential therapies against COVID-19. Our result supports the idea that a systematic approach to repurposing is a valid strategy to accelerate the necessary drug discovery process.

## Abstract (summary)

**Table:** 

Measurement(s)	Cytopathic Effect
Technology Type(s)	confocal fluorescence microscopy
Factor Type(s)	Cellular toxicity
Sample Characteristic - Organism	Chlorocebus sabaeus
Sample Characteristic - Environment	continuant
Sample Characteristic - Location	Belgium

## Background & Summary

Coronaviruses are enveloped viruses carrying 27 to 31 kb single-stranded positive-sense RNA genomes encoding structural and accessory proteins. Severe acute respiratory syndrome coronavirus 2 (SARS-CoV-2) is a respiratory infection that was first recorded in December 2019 and declared as a pandemic in March 2020. Since the start of 2020, there has been almost 5.5 M deaths worldwide with at least 307 Mio assessed infection cases^1^.

Repurposing of known drugs offers a cost and time-effective alternative to the classical antiviral drug discovery approach, which can take many years and involves high costs. Combining high-performance computing and experimental biology is a useful route for drug repurposing efforts in rare, orphan and challenging diseases^2^ as well as health emergencies^3^. The high number of repurposing-based clinical trials currently active worldwide are part of an unprecedented effort in fighting SARS-CoV-2^4^. In line with these efforts, EXSCALATE4COV (E4C) is an EU H2020-funded emergency project which uses high-performance computing and screening infrastructures to identify clinical candidates and progress mono- or combination therapies against SARS-CoV-2 viral infections^5^. Already in the context of the SARS-CoV-2 pandemic, E4C’s supercomputing platform^6,7^ and high-throughput screening assays have been applied to identify modulators of viral protein function across a panel of targets^8–10^.

**Fig. 1:**
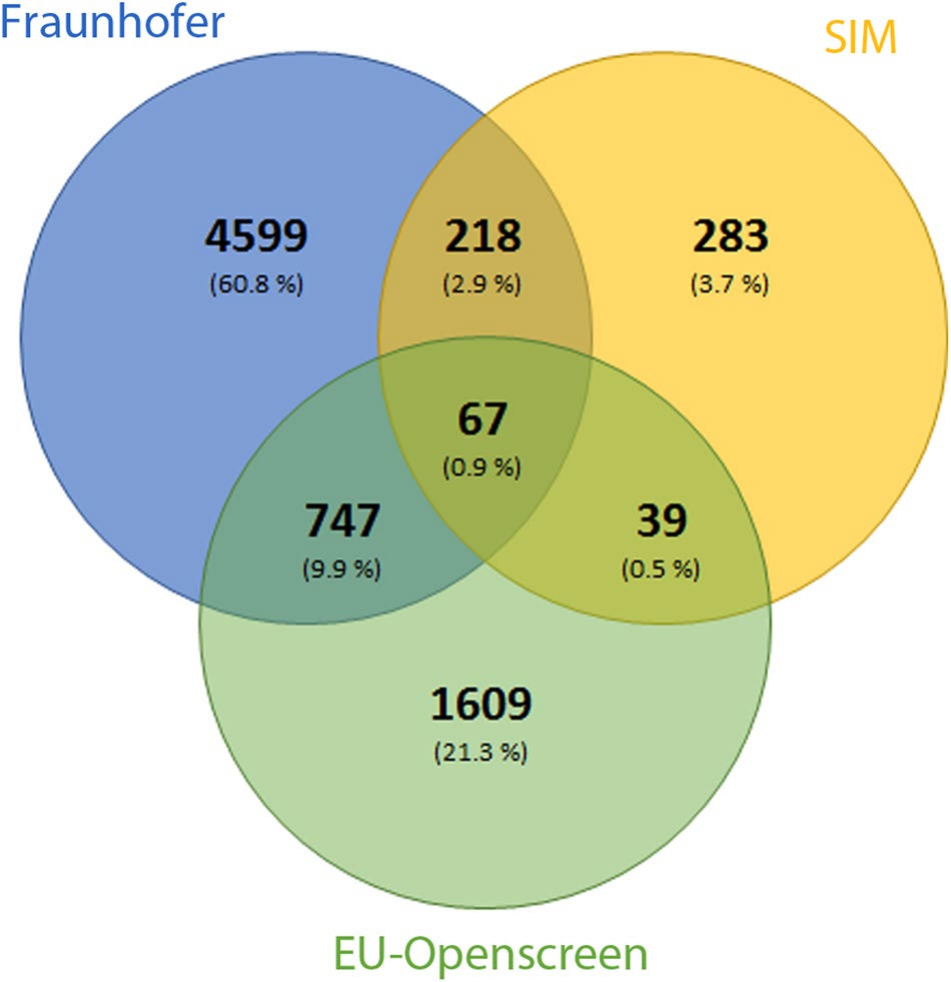
Comparison of the compound collection. Venn diagram showing overlap across the three compound collections in the screened set.

**Fig. 2:**
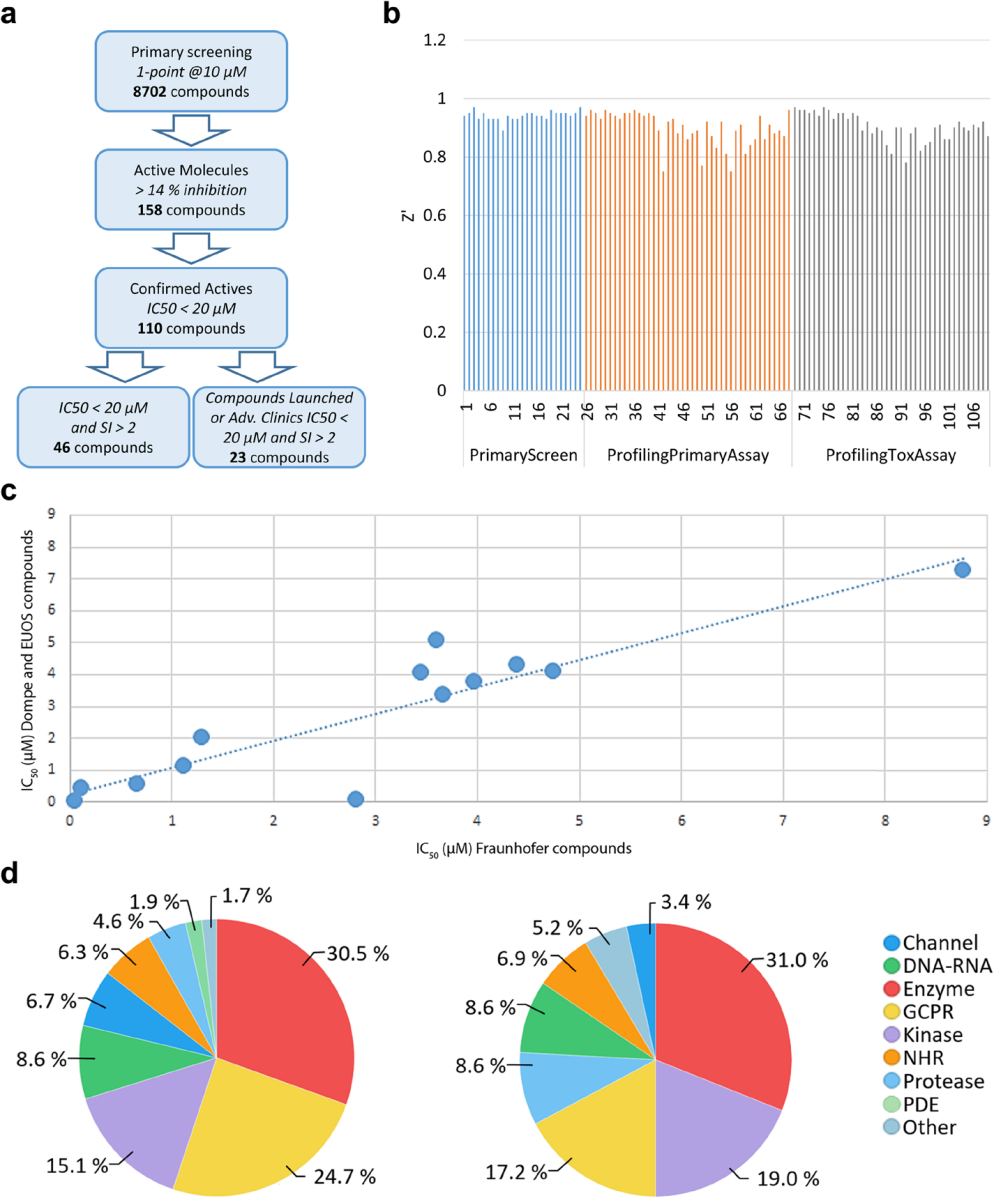
High throughput repurposing screening. (**a**) Workflow and number of compounds at each stage of screening cascade. (**b**) Z’ factor versus screening order for plates across the 3 experimental phases. All values Z’ > 0.5. (**c**) Duplicate compound potencies in hit profiling. IC_50_ values for compounds present in both the Fraunhofer (X axis) and Dompe or EU-OPENSCREEN (Y axis) collections (R^2^ = 0.81). (**d**) Distribution of the primary target of screened compounds (lhs) and 110 confirmed hit compounds (rhs). Explanation of keys: “Channel” gathers all cellular channels comprising metal channels and efflux pumps; “DNA-RNA” comprises all cellular DNA/RNA-dependent enzymes; “Enzymes” gathers a large set of metabolic enzymes involved in cellular metabolism/catabolism mechanisms like de-novo syntheses and/or oxidative or proteolytic processing of non-peptidic substrates; “GPCR” comprises G-Protein coupled receptors; “NHR” stands for Nuclear Hormone Receptors; “Proteases” is self-explaining and “Other” categorizes all the cellular proteins classes not previously listed (e.g. glycosylative enzymes, farnesyltransferase and similar).

The dataset presented in this study was generated from the phenotypic screening of a large scale repurposing collections totalling ca. 8702 compounds (Fig. 1). The primary efficacy assay readout involved fluorescence imaging measurements of compound induced anti-SARS-CoV-2 cytopathic effects on EGFP expressing African green monkey kidney cells (VeroE6-EGFP)^11^. Following primary screening, hit profiling and cytotoxicity assessment, 110 compounds had IC_50_ values below 20 µM and 46 of these have a safety index (ratio of cytotoxic to anti-cytopathic effects) greater than 2 (Workflow shown in Fig. 2). Our results confirmed known, and identified novel repurposed candidates against SARS-CoV-2, including raloxifene (a marketed estrogen receptor antagonist) which, following orthogonal validation studies and independent confirmation by other groups^12^, has been progressed to clinical investigations against COVID-19^13^.

## Methods

### Compound screening set

The screened set was composed of three compound libraries from different sources (Fig. 1). Firstly, the Fraunhofer Repurposing Library contains 5632 compounds including 3,400 compounds that have reached clinical use across 600 indications, as well as 1582 preclinical compounds with varying degrees of validation. This library was assembled by an external partner (SPECS; Netherlands) to mirror a set originally established by the Broad Institute. A curated database is available listing the compounds, indications, primary targets (where known) and mechanism of action, as well as analysis tools that can help to determine the mechanism of action and target. Secondly, the EU-OPENSCREEN Probe Library is a collection of 2500 compounds sampled from launched, clinical and preclinical studies aimed to cover multiple therapeutically relevant cellular mechanisms^14^. Finally, the Dompe’ Farmaceutici S.p.A. proprietary library is a collection of ca 700 candidate drugs which have completed at least phase I clinical trials. All compounds (Table 2) were quality controlled by liquid chromatography/ mass spectrometry (LC/MS) for purity and identity (minimum purity > 90%). The compounds were stored at a concentration of 10 mM in 100% DMSO at –20 °C.

### Cell and virus culture

The African green monkey kidney cell line (Vero E6) was previously engineered to constitutively express GFP^15^. Cells were maintained in Dulbecco’s modified Eagle’s medium (DMEM; Gibco) supplemented with 10% v/v foetal calf serum (FCS; Biowest), 0.075% Sodium Bicarbonate (7.5% solution, Gibco) and 1x Pen-strep (Gibco) and kept under 5% CO2 on 37 °C. Assay medium contained 2% FCS. SARS-CoV-2 strain BetaCov/Belgium/GHB-03021/2020 recovered from a nasopharyngeal swab taken from an asymptomatic patient returning from Wuhan, China in the beginning of February 2020 was sequenced on a MinION platform (Oxford Nanopore). After serial passaging on Huh7 and Vero E6 cells, infectious content of the virus stock was determined by titration on Vero E6 cells using the Spearman-Kärber method. All virus-related work was carried out in certified, high-containment biosafety level-3 facilities in the Rega Institute at KU Leuven.

### Primary anti-cytopathicity assay development: 384-well plates

**Fig. 3:**
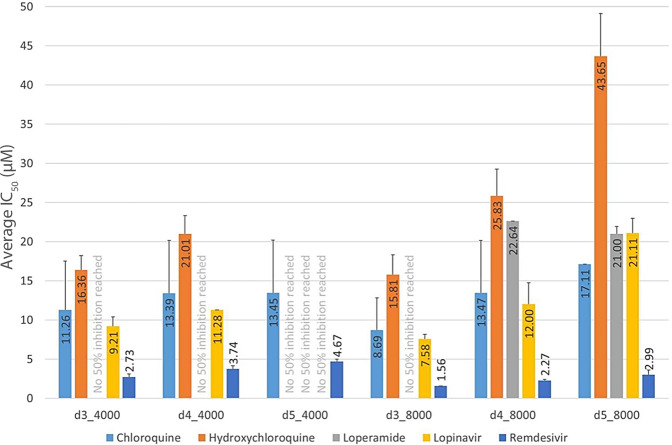
Control compound profiling. Five control compounds were tested: chloroquine (light-blue); hydroxychloroquine (orange bars); loperamide (grey); lopinavir (yellow) and remdesivir (blue). The IC_50_ was measured and reported on the y-axis at different days of incubation. The days of incubation (d) and the number of cells seeded (4000 or 8000) were reported on the x-axis. Data are reported as the mean.

**Fig. 4:**
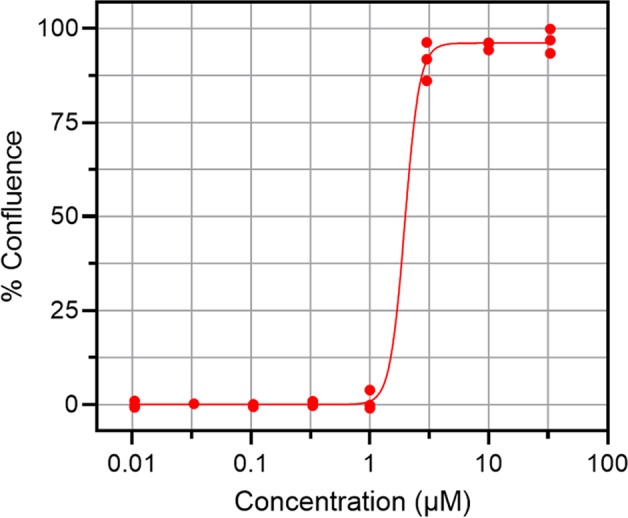
Remdesivir curve fitting example. Performance of positive control. %Confluence versus compound concentration for Remdesivir, IC50 = 1.7 µM, in accord with literature^10^.

The primary assay protocol was optimised with respect to cell seeding, viral MOI and pharmacological response. Cells were seeded at 4000 and 8000 cells/well in 384-well plates. The following day, cells were incubated with the control compounds and the virus at different MOI (0.01 and 0.001). A panel of control compounds was tested to evaluate the pharmacological performance of the assay. Compounds tested were: chloroquine, hydroxychloroquine, and loperamide at a starting concentration of 50 µM; lopinavir and remdevisir at a starting concentration of 100 µM. A dose response curve was achieved with serial dilution at seven different concentration points following a half-log dilution schema. Remdevisir was the most active reference compound under all experimental conditions^16^, (Fig. 3) and was selected as the positive control used at 20 µM final concentration in the primary assay. Positive control remdesivir showed IC50 comparable to literature^10^ as depicted in Fig. 4.

### Hit identification and compound profiling

To measure inhibition of the SARS-CoV-2 cytopathic effect, 384-well imaging plates (Greiner #781092) were spotted with test compounds and controls (16 positive and 16 negative control wells per plate) using an acoustic dispenser (Echo, Labcyte) to yield 10 µM final test compound concentration at 0.1% vol/vol DMSO. For concentration response studies, eight concentrations of a semi-log dilution series from 33 µM to 10 nM at 0.33% DMSO were used. One day before infection (Day -1), test plates were equilibrated to room temperature and 30 µL of Vero E6 EGFP cells were added at 8,000 cells/well. On the day of infection (Day 0), plates were transported to the CAPS-IT robotic system for the addition of virus (MOI = 0.001) using a liquid handler (EVO 100, Tecan) to a final volume of 60 µl, and incubated at 37 °C, 5% CO2 for 4 days. Plates were then imaged on an Arrayscan XTI, (Thermofisher). Parallel assessments of the underlying cytotoxicities of the compounds were performed as described above in dose response studies, but without virus infection and using sodium-selenite (20 µM final) as the intra-plate positive cytotoxicity control.

### Image acquisition and analysis

At day four post-infection, the GFP signal was captured using wide field fluorescence imaging by exciting at 485–20 nm and emitting with the BGRFRN filter set. A 5 X objective captured 80% of the well bottom area in the 384 plate. The optimal exposure time was determined based on fluorescence intensity and was set as 0.023 seconds. A 2 × 2 binning was used and autofocus plane count was reduced to increase image acquisition speed. An image analysis protocol was developed in-house by using the SpotDetector bioapplication (Cellomics, Thermofisher). After background reduction on the raw image files, a fixed fluorescent intensity threshold was determined for the identification of fluorescent cells and their morphologoical parameters were then determined. The two most relevant extracted parameters describing the anticytopathic effects of compounds cells were: i) the number of fluorescent cells in each well (NumberOfCells); and ii) the area of each well covered by fluorescence cells (CellAreaMean).

Test compound results were normalised relative to the corresponding intra-plate controls. For cytopathicity experiments the positive control (100% inhibition of virus induced cytopathicity) were 16 remdesivir (20 µM) containing wells per 384 well plate in column 24. For cytotoxity experiments the positive control (100% cytotoxicity) were 16 sodium-selenite (20 µM) containing wells in column 24. The negative controls (0% effect) for both cytotoxicity and anti-cytopathic experiments were 16 wells with DMSO (0.1% vol/vol) in column 23. The normalised value of “CellAreaMean” was termed “% Confluence” whilst the normalised value of “NumberOfCells” was termed “% Inhibition”. Although, it might be expected that number of cells in each well would scale linearly with the area of the well covered by the cells, compound exposure can also induce changes in cell morphology and dimensions due to poly-pharmacological effects unrelated to any anti-viral properties. Therefore, whilst both parameters are of interest and are made available for re-analysis in the raw data sets provided, the parameter “% Confluence” was used for the purposes of reporting compound effects in ChEMBL and in subsequent compound selection and prioritisation.

Large scale data analysis for Primary and Hit Profiling studies was performed in two ways. The first method used commercial software (ActivityBase, IDBS, Version 8.0.5.4) in a procedure which was aligned with in-house data management policies. Hit profiling dose response data (% Confluence versus compound concentration) were fitted to 4-parameter logistic functions to give the IC_50_ for the anti-cytopathic effect or the CC_50_ for the cytoxicity effects in the absence of virus. Assay quality was assessed using the Z′ factor calculation (Fig. 2B) with Z’ factor > 0.5 as the threshold for assay acceptance^17^. The cytotoxity index was defined as CC_50_/IC_50._ A higher CI value indicates a wider window between the anti-cytopathic effects and possible underlying in-vitro toxicity. Individuals seeking to reanalyse the data may not have access to the ActivityBase software, therefore, a second method was established in the KNIME environment to calculate IC_50_ values from the raw data. The workflow is deposited in github repository and replicates the ActivityBase analysis^18^.

## Data Records

The analysed data and raw data have been made available for download and reuse (Table 1). An overview of all analysed Primary Screening and Hit Profiling data in the ChEMBL repository can be found in the document report card CHEMBL4495565, whilst individual data sets are available for viewing and download from four assay report cards (Table 1). Raw data files for Primary and Hit Profiling have also been made available by the ChEMBL administrators on a FTP server and through the Figshare platform. The variables present in the tables of ChEMBL entries and the raw data files are defined in Table 2.

**Table 1:** Description and location of data records.

Description	Reference
ChEMBL Document Report Card for the complete study	Data available^48^.
Single concentration primary screen for anti-cytopathic effect of compounds (Confluence, %) in ChEMBL DB	Data available^49^.
Hit profiling results for compound anti-cytopathic effect (IC_50_) in ChEMBL DB	Data available^48^.
Hit profiling results for compound cytotoxic effect (CC_50_) in ChEMBL DB	Data available^50^.
Derived cytotoxity index results (CI) in ChEMBL DB	Data available^51^.
Data from the primary screen and hit profiling deposited on CHEMBL FTP server	All screening results in separate files^52^.
Figshare record with figures, tables and Primary data sets available for download	Data can be found at^53–55^

**Table 2:** List of files and variables contained^46,56^. **2a**: Metadata information for ChEMBL document report card CHEMBL4495565^57^. **2b**: Data file for primary and hit profiling raw data (*20201217_primary_PS_HP.xlsx*), variables and descriptions. Table 2b contains the information of the Primary Screen sub-table^49^. A KNIME workflow provided in the code availability section generated the curve fit metrics^17^. **2c**: Data file for primary and hit profiling raw data (*20201217_primary_PS_HP.xlsx*), variables and descriptions. Table 2c contains the information of the Hit Profiling sub-table^49^. **2d**: Data file for primary and hit profiling raw data (*20201217_primary_PS_HP.xlsx*), variables and descriptions. Table 2d contains the information of the Hit Profiling Fit Results sub-table^49^. **2e**: Data file for activity information (*ACTIVITY.tsv*), variables and descriptions^49^. **2f**: Data file for assay information (*ASSAY.tsv*), variables and descriptions^49^. **2g**: Data file for compound record lists (*COMPOUND_RECORD.xlsx*), variables and descriptions^49^. **2h**: Data file for Dose_Response_raw_data (*ACTIVITY_SUPP.tsv*), variables and descriptions^49^. **2i**: Data file for Activity map (*ACTIVITY_SUPP.MAP.tsv*), variables and descriptions^49^. **2j**: Data file for assay reference text (*REFERENCE.tsv*), variables and descriptions^49^.

Metadata name	Metadata content
AssayID	CHEMBL4513082
Type	Functional
Description	Antiviral activity determined as inhibition of SARS-CoV-2 induced cytotoxicity of VERO-6 cells at 10 µM after 48 hours exposure to 0.01 MOI SARS CoV-2 virus by high content imaging
Format	BAO_0000218
Journal	Tbd
Organism	Chlorocebus sabaeus
Strain	—
Tissue	—
Cell Type	Vero C1008
Subcellular Fraction	—
Target	CHEMBL4303835
Document	CHEMBL4495565
Cell	CHEMBL4295411

Variable	Unit	Explanation
Plate Id	dimensionless	Unique Plate identifier.
Well Reference	dimensionless	Well Identifier (Composite Row/Column)
Compound Id	dimensionless	Compound Identifier
CompoundName	dimensionless	Trivial compound name
InChi	dimensionless	InChi structural value
Concentration [µM]	micromolar	Compound concentration used for primary screen
Row	dimensionless	Plate row position identifier
Column	dimensionless	Plate column position identifier
NumberOfCells	dimensionless	Number of fluorescent cells identified in image analysis (variable identical to variable “ValidObjectCount” in HitProfiling subtable)
CellAreaMean	dimensionless	total amount of surface covered by fluorescent cells in image (variable identical to variable “SpotTotalAreaCh2” in HitProfiling subtable)
IntensityCell	dimensionless	Total fluorescence intensity measured in image (variable identical to variable “SpotTotalIntenCh2” in HitProfiling subtable)
% Confluence	percentage	Percentage effect of “CellAreaMean” relative to positive and negative controls
% Inhibition	percentage	Percentage effect of “NumberOfCells” relative to positive and negative controls
WellType	dimensionless	WellType identifier (Compound, DMSO CONTROL, REMDESIVIR CONTROL, EMPTY WELL)

Variable	Unit	Explanation
Compound Id	dimensionless	Compound Identifier
MotherplateID	dimensionless	Unique identifier of compound storage plate
Name	dimensionless	Trivial compound name
InChi	dimensionless	InChi structural value
Well Reference	dimensionless	Well Identifier (Composite Row/Column)
Concentration [µM]	micromolar	Compound concentration used for primary screen
Row	dimensionless	Plate row position identifier
Column	dimensionless	Plate column position identifier
ValidObjectCount_PrimaryAssay	dimensionless	Raw value of number of fluorescent cells identified in image analysis (variable corresponds to variable “NumberOfCells” in Primary screen subtable)
SpotTotalAreaCh2_PrimaryAssay	dimensionless	Raw value of total amount of surface covered by fluorescent cells in image (variable corresponds to variable “CellAreaMean” in PrimaryScreen subtable)
SpotTotalIntenCh2_PrimaryAssay	dimensionless	Raw value of total fluorescence intensity measured in image (variable corresponds to variable “IntensityCell” in Primary screenSubtable)
% Confluence _Primary Assay	percentage	Percentage effect of “CellAreaMean” relative to positive and negative controls in Primary screen
% Inhibition_Primary Assay	percentage	Percentage effect of “NumberOfCells” relative to positive and negative controls determined in Primary screen
ValidObjectCount_ToxAssay	dimensionless	Raw value of number of fluorescent cells identified in image analysis in cytotoxicity assay
SpotTotalAreaCh2_ToxAssay	dimensionless	Raw value of total amount of surface covered by fluorescent cells in image in cytotoxicity assay
SpotTotalIntenCh2_ToxAssay	dimensionless	Raw value of total fluorescence intensity measured in image in cytotoxicity assay
% Confluence_ToxAssay	percentage	Percentage effect of “SpotTotalAreaCh2_ToxAssay” relative to positive and negative controls
% Inhibition_ToxAssay	percentage	Percentage effect of “ValidObjectCount_ToxAssay” relative to positive and negative controls
WellType	dimensionless	WellType identifier (COMPOUND, DMSO CONTROL, REMDESIVIR CONTROL, EMPTY)

Variable	Unit	Explanation
Compound Id	dimensionless	Compound Identifier
Type	dimensionless	Identifier of result type. It can be “IC50”, “CC50”, “SI”, “BOTTOM”, “TOP”, “SLOPE”
Relation	dimensionless	Relation between result type and outcome. It can be “ = ”, “ > ”, “ < ”
Value	various	Result value
Workflow	dimensionless	Identifier of workflow used to generate result. It can be “KNIME” or “ActivityBase”
Unit	dimensionless	Identifier of result unit. It can be “micromolar”, “percent”, “dimensionless”

Variable	Unit	Explanation
ACT_ID	dimensionless	Activity index text collating type of activity measured and progressive index to compound (ACT_INH_001 = primary screen inhibition at fixed concentration for compound 1)
CIDX	dimensionless	Compound Identifier
AIDX	dimensionless	Internal code for screening performed: 1_LEY = primary screening (inhibition); 2_LEY = hit profiling (IC50) 3_LEY = cytotoxicity (CC50) 4_LEY = Selectivity Index (CC50/IC50)
RIDX	dimensionless	Code for screening. In this case LEY_VERO
Type	dimensionless	Description of the data type, could be ‘Inhibition’, ‘Selectivity index’, ‘CC50’ or ‘IC50’
Relation	dimensionless	Relation between data type and outcome. It can be “ = ”, “ > ”, “ < ”
Value	dimensionless	Real number
Upper value	dimensionless	None given
Units	dimensionless	Selectivity index is dimensionless; IC_50_ or CC_50_ values are given in microMoles and inhibition is given in %.
Text_value	dimensionless	None given
Activity_comment	dimensionless	None given
CRIDX	dimensionless	Code for screening. In this case LEY_VERO

Variable	Unit	Explanation
AIDX	dimensionless	Internal code for screening performed: 1_LEY = primary screening (inhibition); 2_LEY = hit profiling (IC50) 3_LEY = cytotoxicity (CC50) 4_LEY = Selectivity Index (CC50/IC50)
RIDX	dimensionless	Code for screening. In this case LEY_VERO
ASSAY DESCRIPTION	dimensionless	Free text describing the content of AIDX
ASSAY_TYPE	dimensionless	Text code for assay type F = functional, T = toxicology
ASSAY_TEST_TYPE	dimensionless	Text code for assay conditions: in this case = *in vitro*
ASSAY_ORGANISM	dimensionless	Organism source according to BAO ontology: in this case = “Chlorocebus sabaeus” for green monkey
ASSAY_STRAIN	dimensionless	None given
ASSAY_TAX_ID	dimensionless	Taxonomy ID: in this case = 60711
ASSAY_SOURCE	dimensionless	None given
ASSAY_TISSUE	dimensionless	None given
ASSAY_CELL_TYPE	dimensionless	Cell type name: in this case = VERO-E6
ASSAY_SUBCELLULAR_FRACTION	dimensionless	None given
TARGET_TYPE	dimensionless	Description of target: In this case = “Organism” for primary screen and for IC50 or = “Cell Line” for cytotoxicity in absence of virus
TARGET_NAME	dimensionless	Target name: In this case = “Severe acute respiratory syndrome coronavirus 2” or = VERO-E6
TARGET_ACCESSION	dimensionless	None given
TARGET_ORGANISM	dimensionless	None given

Variable	Unit	Explanation
CIDX	dimensionless	Compound Identifier
RIDX	dimensionless	Code for screening. In this case LEY_VERO
COMPOUND_NAME	dimensionless	Compound Trivial Name
COMPOUND_KEY	dimensionless	Compound Trivial name or IUPAC

Variable	Unit	Eplanation
SAM_ID	dimensionless	Sample index text collating type of activity measured and progressive index to compound (SAM_CC50_001 = measurements target (IC50 or CC50) for compound 1)
TYPE	dimensionless	Text describing collating type of value type (concentrations or readouts) and type of experiment
RELATION	dimensionless	Relation between data type and outcome. It can be “ = ”, “ > ”, “ < ”
VALUE	dimensionless	Real number
UNIT	dimensionless	Readout values are given in µM and inhibition values are given in %.
REG_ID	dimensionless	Text collating type of experiment with index of molecules and index of concentrations (all in triplicates)

Variable	Unit	Explanation
ACT_ID	dimensionless	Activity index text collating type of activity measured and progressive index to compound (ACT_INH_001 = primary screen inhibition at fixed concentration for compound 1) (see above ACTIVITY.XLSX)
SAM_ID	dimensionless	Sample index text collating type of activity measured and progressive index to compound (SAM_CC50_001 = measurements target (IC50 or CC50) for compound 1)

Variable	Unit	Explanation
RIDX	dimensionless	Code for screening. In this case LEY_VERO
PUBMED_ID	dimensionless	Future PUBMED_ID (link for accepted paper)
JOURNAL_NAME	dimensionless	Journal Name where paper is or will be published
YEAR	dimensionless	Year of publication
VOLUME	dimensionless	Journal Volume where paper is or will be published
ISSUE	dimensionless	Journal Issue where paper is or will be published
FIRST_PAGE	dimensionless	Integer for first page where paper is or will be published
LAST_PAGE	dimensionless	Integer for last page where paper is or will be published
REF_TYPE	dimensionless	Reference type: Dataset
TITLE	dimensionless	Cytopathic SARS-CoV-2 screening on VERO-E6 cells in a large-scale repurposing effort
DOI	dimensionless	To be given by Editor/Publisher
PATENT_ID	dimensionless	None given
ABSTRACT	dimensionless	Text of paper abstract
AUTHORS	dimensionless	Authors’ list

## Technical Validation

### Screening Assay

The primary screen resulted in 158 hits (% confluence > 14%) (CHEMBL4495565 – Fig. 2a). At all phases (Primary, Profiling and Cytotoxicity) the Z’ exceeded > 0.5, indicating acceptable assay quality (Fig. 2b). Sources of compounds from the three different libraries used in the screening campaign are shown in Table 3. Primary hit compounds were cherry-picked and progressed to dose response profiling and cytotoxicity assessment. In profiling studies, identical compounds present in different libraries (Fig. 1) showed consistent potency (Fig. 2c,R^2^ = 0.81), suggesting good assay reproducibility with respect to compound origin. Some 110 compounds gave IC_50_ values below 20 µM and were classified as confirmed hits. From the 110 compounds, 66 have a SI > 2 (Supp. Table S1 and Fig. 2a) and of this group 18 compounds are either marketed drugs or in advanced clinical trials (Fig. 2a). (CHEMBL449565). Fig. 2d shows the distribution of primary therapeutic targets annotated for the screened collection (lhs) and the 110 confirmed hits (rhs). No changes in the targets of the confirmed hits relative to those of the screened compound set were observed.

**Table 3:** Source of compounds from the three different libraries used in the screening campaign.

Compounds Provenance	No. Compounds	Reference
Fraunhofer Repurposing Library	5632	Data available^58^.
EU_OPENSCREEN Bioactive set	2500	Data available^59^.
DOMPE_SIM	700	Data available^60^.

### Assay properties and influence on hit compound identification

SARS-CoV infects and replicates more efficiently in some cell types, such as VeroE6, FRhK4, Caco2, LLCMK2, compared to Calu3 and Hek293T cells, while there is a very low efficiency of replication in U251 and MDCK cells under the same multiplicity of infection. Therefore, cell model selection in SARS-COV-2 phenotypic screening studies is important, and recent reports have shown differences in compound potencies depending which cell line has been used in the primary assay^19–27^. The cell line used in this study, Vero E-6 kidney epithelial cells from the African green monkey^28^, has been extensively used for SARS-CoV-like virus studies^29–33^. In those models, cell viability and virus titre were usually verified after 3–5 days post infection (p.i.)^27,34^ and in our assay the incubation of compounds for a period of 4 days p.i. resulted in a robust readout. Nevertheless, it has already been reported that for the same active drug, infection with different virus MOI may result in variable safety index values^35^, suggesting that cytotoxicity analyses in all studies could be somewhat limited. It should also be noted that the VeroE6-EGFP cell line used for this study is not sensitive to ACE inhibitors or some SARS-CoV active drugs such as ribavirin and glycyrrhizin^36–39^, which are the subject of ongoing clinical trials We cannot exclude, therefore, that alternative experimental setups may lead to partially different hit populations, as others have observed^19^. Nevertheless, the GFP reporter line presents an opportunity to perform fast, automated and homogeneous high-throughput screening, with a high signal-to-noise ratio and low variability and is fit-for-purpose for hit identification studies.

Within the confirmed compounds with IC_50_ < 20 µM, some 70% modulate the intracellular signalling pathways. Notable groups are the inhibitors of the Growth Factor Receptors (PDGFR) (like masitinib or tandutinib), Dihydrofolate Reductase (DHFR) (trimetrexate) and Estrogen Receptor Modulator (clomiphene and raloxifene). In addition, many show common protein targets such as the Phosphatidylinositol 3-Kinase (PI3K) (VPS34-IN-1) or mTOR protein (VE-822) two key elements of pro-survival signalling. Finally, where a therapeutic indication was annotated, the majority of the compounds were associated with cancer and anti-infective (antifungal and anti-malaria) therapy. These observations suggest that drugs associated with cell survival and growth may be an optimal choice for antiviral therapies for SARS-CoV-2, if adequate safety and exposure/efficacy can be achieved. Comparing the hit population in this dataset with reported studies show five compounds (amodiaquine, ciclesonide, eltrombopag, loperamide, niclosamide) that are also reported by Jeon *et al*., who screened 3000 FDA approved drugs against Vero cells^15^. Similarly, two (amodiaquine, chlorpromazine) overlap with a set of 20 inhibitors identified by Weston^29^, also in Vero cells. The common compounds have an antifungal, antimalarial or anticancer activity, suggesting again that drugs for these indications may contain the most promising antiviral compounds. Among compounds selected for further studies, raloxifene was prioritised as it has been found active in an independent phenotypic assays of SARS CoV-2 viral infections in VERO-E6 cells^40^ and against coronavirus OCR43 in LLC-MK2 cells^41^. This compound can inhibit RNA replication^42^ and related estrogenic receptor modulators have been found to be active against in-vitro viral infections^43^. Interestingly, there have been evidence from other datasets^44^ that several SERMS, either agonist or antagonists, are active at SARS-CoV 2 viral entry level and we are also exploring this and other possible mechanisms of action for this class of compounds.

## Usage Notes

We suggest that other similar screening data sets are available from the Covid-19 portal^45^, the NCATS databases^46^ and a newly developed collection called “The COVID-19 Drug and Gene Set Library”^47^.

## Supplementary information

Supplementary Table 1

## Data Availability

We developed a more comprehensive open-source KNIME workflow for the curve fitting calculations^17^.
