# Supplementary material for: Cytopathic SARS-CoV-2 screening on VERO-E6 cells in a large-scale repurposing effort
Source: Sci Data. 2022 Jul 13;9:405. doi: 10.1038/s41597-022-01532-x (PMC9279437; doi:10.1038/s41597-022-01532-x)
Supplement: Supplementary file 1 — Supplementary Table 1 [file 41597_2022_1532_MOESM1_ESM.docx]

**Supporting Table 1**: Selected active compounds with IC_50_ <20µM and SI>2.

| **Name** | **IC50 (µM)** | **CC50 (µM)** | **SI (CC50/IC50)** | **Phase** | **Target** |
| --- | --- | --- | --- | --- | --- |
| Hypericin | 0.25 | 1.31 | 5.24 | Phase 1 | Cholesteryl Ester Transfer Protein (CETP) Inhibitors; HDL-Cholesterol Increasing Agents |
| MG-132 | 0.32 | 2.90 | 9.06 | Preclinical | PSMB1 |
| Amrubicin | 0.33 | 33.00 | 100.00 |  |  |
| Trimetrexate | 0.33 | 1.99 | 6.03 |  | FOLA |
| Coumarin 7 | 0.36 | 33.00 | 91.67 |  | RAB9A |
| VPS34-IN-1 | 0.40 | 26.83 | 67.08 |  | PIK3C3 |
| Fluorescein | 0.67 | 33.00 | 49.25 | Launched | SLC22A6 |
| Rhodamine-123 | 0.80 | 33.00 | 41.25 | Phase 1 |  |
| Cycloheximide | 1.16 | 10.16 | 8.76 |  | SLC2A1 |
| VE-822 | 2.67 | 10.02 | 3.75 | Phase 2 | ATM, ATR, MTOR, PIK3CG |
| N-tert-Butylisoquine | 3.43 | 12.43 | 3.62 | Phase 1 | Apoptosis Inducers; Microtubule Destabilizers (Tubulin Polymerization Inhibitors); Antimitotic Drugs |
| DHCaA | 3.69 | 15.68 | 4.25 |  | AHCY |
| 8-Azaguanine | 3.72 | 10.38 | 2.79 |  | LOX1.5 |
| CP-640186 | 3.78 | 10.22 | 2.70 |  | ACACB |
| Bepridil | 3.86 | 23.44 | 6.07 | Launched | ATP1A1, CACNA1A, CACNA1H, CACNA2D2, CALM1, KCNQ1, KCNQ4, PDE1A, PDE1B, TNNC1 |
| Thaliblastine | 3.87 | 33.00 | 8.53 | Phase 2 |  |
| AEE788 | 3.94 | 33.00 | 8.38 | Phase 1 | AGE Inhibitors (Maillard's Reaction Inhibitors) |
| MMV665852 | 3.97 | 33.00 | 8.31 |  | EPHX1 |
| KD-023 | 4.04 | 33.00 | 8.17 | Phase 2 | ACACA, ACACB |
| Amodiaquine | 4.07 | 16.78 | 4.12 |  | HNMT |
| Niclosamide | 4.10 | 33.00 | 8.05 | Phase 1 | 5-HT2A Receptor Antagonists; Signal Transduction Modulators; Dopamine D2 Receptor (DRD2) Antagonists |
| STF-62247 | 4.11 | 31.50 | 7.66 | Preclinical |  |
| CP-640186 (hydro-chloride) | 4.14 | 10.88 | 2.63 | Preclinical | ACACA, ACACB |
| Tandutinib (MLN518) | 4.23 | 33.00 | 7.80 | Phase 2 | CSF1R, FLT3, KIT, PDGFD, PDGFRB |
| PHA-665752 | 4.97 | 33.00 | 6.64 | Preclinical | MET |
| Thieno-pyridone | 5.26 | 33.00 | 6.27 |  | PTP4A3 |
| Halofantrine hydrochloride | 5.31 | 33.00 | 6.21 | Phase 1 | D-Amino Acid Oxidase (DAAO) Inhibitors |
| Golvatinib (E7050) | 5.89 | 33.00 | 5.60 | Phase 2 | KDR, MET |
| NELFINAVIR MESYLATE | 7.08 | 33.00 | 4.66 | Launched | CYP1A2, CYP2B6, CYP2C19, CYP2C9, CYP2D6, CYP3A4, CYP3A7 |
| 3'-fluoro-benzyl-spiperone | 7.66 | 27.41 | 3.58 | Preclinical | DRD2 |
| Adomeglivant | 10.55 | 33.00 | 3.13 |  | GCGR |
| 6-amino-chrysene | 11.64 | 33.00 | 2.84 |  | HPRT1 |
| VU-0364739 | 11.64 | 33.00 | 2.84 |  |  |
| Tetrandrine (Fanchinine) | 11.66 | 33.00 | 2.83 | Preclinical | SLC6A3 |
| Masitinib (AB1010) | 11.90 | 33.00 | 2.77 | Launched | FGFR3, KIT, PDGFRA, PDGFRB |
| CFI-400945 | 11.91 | 33.00 | 2.77 |  | PLK4 |
| UNC0646 | 12.03 | 33.00 | 2.74 |  | EHMT2 |
| IPAG | 12.76 | 33.00 | 2.59 | Preclinical |  |
| BS-181 | 12.93 | 33.00 | 2.55 | Preclinical | CDK7 |
| MCOPPB | 13.14 | 33.00 | 2.51 |  |  |
| MF63 | 13.29 | 33.00 | 2.48 |  | PTGES |
| Clomiphene citrate | 13.62 | 33.00 | 2.42 | Launched | ESR1 |
| casin | 14.15 | 33.00 | 2.33 |  |  |
| Raloxifene hydrochloride | 15.44 | 33.00 | 2.14 | Launched | ESR1, ESR2 |
| Thioguanosine | 15.59 | 33.00 | 2.12 | Phase 2 |  |
